# The TOR signalling pathway in fungal phytopathogens: A target for plant disease control

**DOI:** 10.1111/mpp.70024

**Published:** 2024-11-07

**Authors:** Yun Song, Yaru Wang, Huafang Zhang, Muhammad Abu Bakar Saddique, Xiumei Luo, Maozhi Ren

**Affiliations:** ^1^ College of Agriculture and Biology Liaocheng University Liaocheng China; ^2^ Institute of Urban Agriculture, Chinese Academy of Agricultural Sciences; Chengdu Agricultural Science and Technology Center Chengdu China

**Keywords:** disease control, plant pathogens, TOR signalling pathway

## Abstract

Plant diseases caused by fungal phytopathogens have led to significant economic losses in agriculture worldwide. The management of fungal diseases is mainly dependent on the application of fungicides, which are not suitable for sustainable agriculture, human health, and environmental safety. Thus, it is necessary to develop novel targets and green strategies to mitigate the losses caused by these pathogens. The target of rapamycin (TOR) complexes and key components of the TOR signalling pathway are evolutionally conserved in pathogens and closely related to the vegetative growth and pathogenicity. As indicated in recent systems, chemical, genetic, and genomic studies on the TOR signalling pathway, phytopathogens with TOR dysfunctions show severe growth defects and nonpathogenicity, which makes the TOR signalling pathway to be developed into an ideal candidate target for controlling plant disease. In this review, we comprehensively discuss the current knowledge on components of the TOR signalling pathway in microorganisms and the diverse roles of various plant TOR in response to plant pathogens. Furthermore, we analyse a range of disease management strategies that rely on the TOR signalling pathway, including genetic modification technologies and chemical controls. In the future, disease control strategies based on the TOR signalling network are expected to become a highly effective weapon for crop protection.

## Graphical abstract

We discuss the current knowledge on the fungal TOR signalling pathway and analyse a range of disease management strategies that rely on the TOR pathway, which are expected to become a highly effective weapon for crop protection.

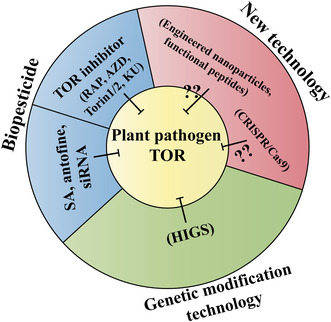

## 1 INTRODUCTION

Plant diseases caused by fungal pathogens directly cause huge losses in the yield of staple crops and economically important commodity crops. Moreover, pathogen infection indirectly exerts a burden on the social, environmental, and economic costs of control (Chaloner et al., 2021). Recently, the change in climate and atmospheric composition has led to emerging pathogens, defined as those that infect new crops and in new places (Fones et al., 2020). The above adverse effects pose significant threats to global food security and reinforce the need to develop new, efficacious, and environmentally sound approaches to control plant disease. The target of rapamycin (TOR) signalling pathway is highly conserved in all major eukaryotic lineages except in some parasites and functions as a major hub controlling growth responses through the integration of nutrients, hormones, and environmental signals (Burkart & Brandizzi, 2021; Henriques et al., 2022; Jamsheer et al., 2022). Over the past decades, extensive research has demonstrated an emerging pivotal role of this pathway in plant disease control. In this review, we delve into a comprehensive analysis of the intricate signalling landscape of the TOR network in phytopathogens, highlighting the specific inhibitors commonly employed to target this pathway. We explore recent breakthroughs in understanding the TOR pathway's pivotal role in promoting phytopathogen growth and proliferation, which are crucial for infection and host colonization. Additionally, we uncover the emerging roles of the plant TOR pathway in mediating host–phytopathogen interactions, shedding light on the complex molecular dialogues that underpin these relationships. Furthermore, we present a detailed examination of innovative disease control strategies that leverage the TOR pathway, including the use of genetic modification technologies to disrupt TOR signalling in pathogens and the development of chemical agents designed to inhibit TOR function. We also discuss the potential of combining these strategies with traditional agricultural practices to enhance their efficacy and sustainability. Despite significant advances, considerable challenges remain in fully harnessing the TOR pathway to manage pathogens in practical crop production effectively. Ongoing research is essential to unravel the complexities of TOR signalling and to develop robust, field‐applicable solutions that can withstand the evolving threats posed by phytopathogens.

## 2 TOR SIGNALLING NETWORK

**FIGURE 1:**
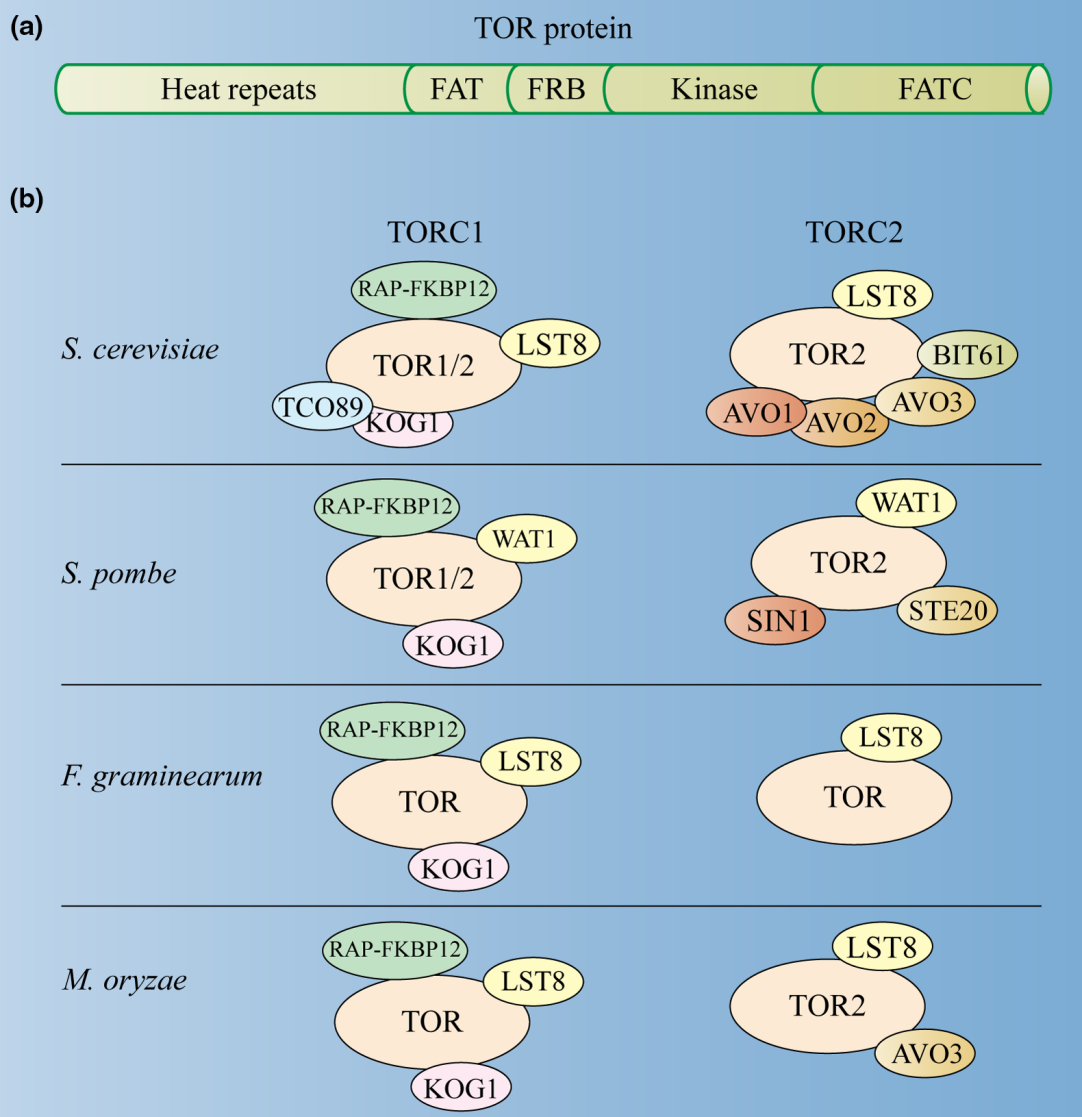
Structures and complexes of TOR kinase in the model fungi *Saccharomyces cerevisiae* and *Schizosaccharomyces pombe*. (a) Conserved domain structure of TOR kinase. TOR kinase domain architecture is highly conserved. TOR contains the N‐terminal clusters of huntingtins, elongation factor 3, a subunit of protein phosphatase 2A and TOR1 (HEAT) repeats, followed by a FRAP, ATM, and TRRAP (FAT) domain; the FKBP12–rapamycin binding (FRB) domain; the catalytic kinase domain; and the C‐terminal FATC domain. (b) Components of TORC1 and TORC2 in *S. cerevisiae*, *S. pombe*, and two well‐studied representative plant‐pathogenic fungi *Fusarium graminearum* and *Magnaporthe oryzae*. Rapamycin (RAP)‐FKBP12 complex binds to the TORC1 complex, instead of the TORC2 complex. AVO1/2/3, adheres stroly (to TOR2) 1/2/3; BIT61, binding partner of TOR2 protein 61; KOG1, kontroller of growth 1; LST8, lethal with SEC. 13 protein 8; SIN1, MAPK‐interacting protein 1; STE20, a homologue of AVO1; TCO89, TOR complex one 89; TOR, target of rapamycin; TORC1, TOR complex 1; TORC2, TOR complex 2; WAT1, a homologue of LST8.

The target of rapamycin (TOR) was first identified in yeast (*Saccharomyces cerevisiae*) by screening mutants insensitive to rapamycin (RAP), which is a medicine isolated from the bacterium *Streptomyces hygroscopicus* and efficiently represses TOR kinase activity (Heitman et al., 1991; Sehgal et al., 1975; Vézina et al., 1975). FK506 binding protein 12 (FKBP12) is a peptidylprolyl isomerase, and functions as a cofactor in rapamycin‐mediated inhibition of TORC1 (Heitman et al., 1991). Further studies revealed that FKBP12 forms a ternary complex with rapamycin and the FKBP12‐rapamycin binding (FRB) domain of TOR to repress its activity. Since then, extensive studies have been carried out to elucidate the mechanisms and functions of TOR in distinct eukaryotic organisms. These studies have demonstrated that TOR serves as a central regulator that coordinates cell growth with light availability, the diurnal cycle, energy availability, and hormonal pathways (Burkart & Brandizzi, 2021; Henriques et al., 2022; Pacheco et al., 2021). TOR protein is an evolutionally conserved Ser/Thr protein kinase and belongs to the phosphatidylinositol 3‐kinase‐related kinase (PIKK) family (Meng et al., 2022). This protein contains five remarkably conserved domains: several HEAT (Huntington, elongation factor 3 regulatory, subunit A of PP2A, TOR1) repeats at the N‐terminal, a FAT (FRAP‐ATM‐TRRAP) domain, an FRB domain, a kinase domain, and a FATC (carboxy‐terminal FAT) domain at the C‐terminal (Figure 1a) (Liu & Xiong, 2022). In most of the eukaryotic lineages, there is only one copy of the *TOR* gene. Yeast and some fungi possess two *TOR* genes.

TOR forms a proteinaceous complex with its primary interactors to exert its function (Figure 1b). In yeast and mammals, TOR forms two structurally and functionally distinct proteinaceous complexes: TOR complex 1 (TORC1) and TOR complex 2 (TORC2) (Brunkard, 2020). The core components of TORC1 include TOR, the regulatory‐associated protein of mTOR (RAPTOR)/KOG1, and lethal with SEC13 protein 8 (LST8). The TOR protein recruits LST8, SAPK‐interacting protein 1 (SIN1)/AVO1, and rapamycin‐insensitive companion of mTOR (RICTOR)/AVO3 to form TORC2. The TORC1 complex is rapamycin‐sensitive, while the TORC2 complex is insensitive to rapamycin (Brunkard, 2020). The upstream regulation signals sensed and downstream targets controlled by TOR complexes among diverse eukaryotic organisms have been thoroughly reviewed in numerous excellent studies (Brunkard, 2020; Burkart & Brandizzi, 2021; Eltschinger & Loewith, 2016; Jamsheer et al., 2022; Liu & Sabatini, 2020; Liu & Xiong, 2022; Shimobayashi & Hall, 2014). Therefore, only selected components of the TOR signalling landscape will be mentioned below in this review.

Rapamycin, a secondary metabolite produced by *S. hygroscopicus*, is named from its place of origin and can efficiently repress TOR kinase activity (Heitman et al., 1991; Sehgal et al., 1975; Vézina et al., 1975). As its exquisite selectivity for TOR, rapamycin exhibits its antifungal, immunosuppressive, and anticancer properties and is widely used in TOR studies of various organisms including yeasts and mammals. Subsequent studies revealed the lack of distinction between the performance of rapamycin and its analogues and thus promoted the development of the second generation of TOR inhibitors asTORis (active‐site TOR inhibitors) (Benjamin et al., 2011; Dong et al., 2015; Zhang et al., 2011). The asTORis are ATP analogues which compete with ATP for binding to the TOR kinase domain. Thus, compared with rapamycin, asTORis can efficiently hinder the activity of both TORC1 and TORC2. Recently, asTORis including AZD‐8055 (AZD), Ku‐0063794 (KU), Torin1, and Torin2 has been successfully applied to dampen TOR activity (Benjamin et al., 2011; Dong et al., 2015; Li et al., 2021).

## 3 TOR SIGNALLING LANDSCAPE IN PHYTOPATHOGENS

Recent studies revealed that rapamycin treatment could also effectively hinder TOR protein activity in phytopathogens, suggesting that the TOR signalling pathway also exists and is evolutionarily conserved in phytopathogens (Bastidas et al., 2012; López‐Berges et al., 2010a; Meléndez et al., 2009; Teichert et al., 2006; Yu et al., 2014). For now, we find the published research about the phytopathogen TOR pathway mainly focuses on that in phytopathogenic fungi. As such, this review is primarily concerned with the progress of the TOR signalling pathway made in phytopathogenic fungi. Based on previous studies and scientific/economic importance (Dean et al., 2012; Haas et al., 2009; Tatebe & Shiozaki, 2017), this review mainly investigates the TOR signalling pathway in 10 fungal pathogens: *Botrytis cinerea*, *Blumeria graminis* f. sp. *tritici*, *Colletotrichum graminicola*, *Fusarium graminearum*, *Fusarium oxysporum*, *Magnaporthe oryzae*, *Phytophthora infestans*, *Verticillium dahliae*, *Puccinia striiformis* f. sp. *tritici*, and *Ustilago maydis*. As for the availability of the wealth of the sequenced genomes, we conduct comparative genomic studies to annotate the TOR pathway in the fungal kingdom. To identify the evolutionarily conserved TOR signalling pathway components in fungal pathogens, the amino acid sequences of corresponding components of the TOR signalling pathway for *Saccharomyces cerevisiae*, *Schizosaccharomyces pombe*, *Homo sapiens*, and *Arabidopsis thaliana* were used to BLAST search for the homologous sequences in the genomes of the 10 fungal pathogens mentioned above (Table 1, Table S1). As predicted, the conserved TOR signalling pathways exist in phytopathogenic fungi. In most of the investigated fungal genomes, there is only one copy of the *TOR* gene. Two *TOR* genes are present in the genomes of *F. oxysporum* and *P. infestans*. Duplications of the TOR protein in these two species may result from independent segmental gene duplication events or whole genome duplication events (Shertz et al., 2010). Alignment of the TOR protein sequences from phytopathogenic fungi with those from *S. cerevisiae* and *S. pombe* showed similar conserved domain organization and high identification. In contrast, the FATC domain was not identified in the *Fo*TOR2 (Figure S1A). The kinase domain sequence alignment and phylogenetic analysis further indicate that the TOR protein of phytopathogenic fungi is evolutionarily conserved (Figure S1B,C). The homologous proteins of the components of TORC1 are found in all the investigated fungi. Meanwhile, except for *F. graminearum*, putative homologues encoding specific components of TORC2, including TOR, LST8, and RICTOR, are present in the genome of the other pathogens. The proteins SIN1/AVO1 are absent from the genome of *C. graminicola*, *F. graminearum*, *M. oryzae*, *P. infestans*, and *P. striiformis* f. sp. *tritici*, indicating that SIN1/AVO1 may play less crucial parts or be replaced by some other components in the evolutionary history and life strategies of these pathogens.

**TABLE 1:** Putative components of TOR signalling network in diverse phytopathogenic fungi.

Protein name	Sc	Sp	Hs	At	Bc	Bgt	Cg	Fg	Fo	Mo	Pi	Pst	Vd	Um
TORC1	✓	✓	✓	✓	✓	✓	✓	✓	✓	✓	✓	✓	✓	✓
TOR	✓	✓	✓	✓	✓	✓	✓	✓	✓	✓	✓	✓	✓	✓
LST8	✓	✓	✓	✓	✓	✓	✓	✓	✓	✓	✓	✓	✓	✓
RAPTOR/KOG1	✓	✓	✓	✓	✓	✓	✓	✓	✓	✓	✓	✓	✓	✓
PRAS40			✓											
DEPTOR			✓											
TCO89	✓													
TORC2	✓	✓	✓		✓	✓	✓		✓	✓	✓	✓	✓	✓
TOR	✓	✓	✓	✓	✓	✓	✓	✓	✓	✓	✓	✓	✓	✓
LST8	✓	✓	✓	✓	✓	✓	✓	✓	✓	✓	✓	✓	✓	✓
RICTOR/AVO3	✓	✓	✓		✓	✓	✓		✓	✓	✓	✓	✓	✓
DEPTOR			✓											
mSIN1/AVO1	✓	✓	✓		✓	✓			✓				✓	✓
PROTOR1/2			✓											
AVO2	✓					✓			✓				✓	
Key upstream components														
FKBP12	✓	✓	✓	✓	✓	✓	✓	✓	✓	✓	✓	✓	✓	✓
TSC1/2		✓	✓			✓				✓				✓
Rheb	✓	✓	✓		✓	✓	✓	✓	✓	✓	✓	✓	✓	✓
PLD1	✓	✓	✓	✓	✓	✓	✓	✓	✓	✓	✓	✓	✓	✓
VPS34	✓	✓	✓	✓	✓	✓	✓	✓	✓	✓	✓	✓	✓	✓
AMPK/SNF1	✓	✓	✓	✓	✓	✓	✓	✓	✓	✓	✓	✓	✓	✓
PTEN	✓	✓	✓	✓	✓	✓	✓	✓	✓	✓	✓	✓		✓
PDK	✓	✓	✓	✓	✓	✓	✓	✓	✓	✓	✓	✓		✓
AKT/PKB	✓	✓	✓	✓	✓	✓	✓	✓	✓	✓	✓	✓	✓	✓
VAM6	✓	✓	✓		✓	✓	✓	✓		✓			✓	✓
EGO1/3	✓													
GTR1/2	✓	✓			✓	✓	✓	✓	✓	✓	✓	✓	✓	✓
GATOR1/2	✓	✓	✓		✓	✓	✓	✓	✓	✓	✓	✓	✓	
TTT‐R2TP	✓	✓	✓	✓	✓	✓	✓	✓	✓	✓	✓	✓	✓	
Key downstream components														
S6K1/Sch9	✓	✓	✓	✓	✓	✓	✓	✓	✓	✓	✓	✓	✓	✓
RPS6	✓	✓	✓	✓	✓		✓	✓	✓	✓	✓	✓	✓	✓
ATGs	✓	✓	✓	✓	✓	✓	✓	✓	✓	✓	✓	✓	✓	✓
SFP1	✓	✓	✓	✓	✓		✓		✓	✓		✓	✓	
SIT4	✓	✓	✓	✓	✓	✓	✓	✓	✓	✓	✓	✓	✓	✓
TAP42	✓	✓	✓	✓	✓	✓	✓	✓	✓	✓		✓	✓	✓
YPK2	✓	✓			✓	✓	✓	✓	✓	✓	✓	✓	✓	✓

Abbreviations: At, *Arabidopsis thaliana*; Bc, *Botrytis cinerea*; Bgt, *Blumeria graminis* f. sp. *tritici*; Cg, *Colletotrichum graminicola*; Fg, *Fusarium graminearum*; Fo, *Fusarium oxysporum*; Hs, *Homo sapiens*; Mo, *Magnaporthe oryzae*; Pi, *Phytophthora infestans*; Pst, *Puccinia striiformis* f. sp. *tritici*; Sc, *Saccharomyces cerevisiae*; Sp, *Schizosaccharomyces pombe*; Um, *Ustilago maydis*; Vd, *Verticillium dahliae*.

**TORC1**: DEPTOR, DEP domain containing mTOR interacting protein; KOG1, kontroller of growth 1; LST8, lethal with SEC. 13 protein 8; PRAS40, proline‐rich AKT substrate 40 kDa; RAPTOR, regulatory‐associated protein of mTOR; TCO89, TOR complex one 89; TOR, target of rapamycin; TORC1, TOR complex 1.

**TORC2**: AVO1, adheres voraciously (to TOR2) 1; AVO2, adheres voraciously (to TOR2) 2; AVO3, adheres voraciously (to TOR2) 3; mSIN1, MAPK‐interacting protein 1; PROTOR1/2, protein observed with RICTOR 1 or 2; RICTOR, rapamycin‐insensitive companion of mTOR; TORC2, TOR complex 2.

**Key upstream components**: AKT/PKB, phosphatidylinositol 3 kinase/protein kinase B; AMPK, AMP‐activated protein kinase; EGO1, also known as Meh1; EGO3, also known as Slm4; FKBP12, FK506‐binding protein 12; GATOR1/2, GAP activity towards Rags 1/2; GTR1/2, glucosinolate transporters 1/2; PDK, pyruvate dehydrogenase kinase; PLD1, phospholipase D1; PTEN, phosphatase and tensin homologue; Rheb, Ras homologues, mTORC1 binding; SNF1, sucrose non‐fermenting 1; TSC1/2, tuberous sclerosis complex 1/2; TTT‐R2TP, (Tel2‐Tti1‐Tti2)–R2TP complex; VAM6, a member of the homotypic fusion and vacuole protein; VPS34, vacuolar protein sorting 34.

**Key downstream components**: ATG, autophagy‐related; RPS6, ribosomal protein S6; S6K1, ribosomal protein S6 kinase; SFP1, major facilitator superfamily protein; SIT4, PP2A‐like protein phosphatase; TAP42, two a‐associated protein of 42 kDa; YPK2, the yeast homologue of SGK1 (serum‐ and glucocorticoid‐activated kinase).

**Genome database**: Bc: http://fungi.ensembl.org/Botrytis_cinerea/Info/Index; Bgt: https://fungi.ensembl.org/Blumeria_graminis_f_sp_tritici_gca_900519115/Info/Index; Cg: https://fungi.ensembl.org/Colletotrichum_graminicola/Info/Index; Fg: https://fungi.ensembl.org/Fusarium_graminearum_ph_1_gca_000240135/Info/Index; Fo: http://fungi.ensembl.org/Fusarium_oxysporum/Info/Index?db=core; Mo: https://fungi.ensembl.org/Magnaporthe_oryzae/Info/Index; Pi: https://www.ncbi.nlm.nih.gov/bioproject/17665; Pst: https://fungi.ensembl.org/Puccinia_striiformis_f_sp_tritici_pst_78_gca_001191645/Info/Index; Vd: https://fungi.ensembl.org/Verticillium_dahliaejr2/Info/Index; Um: https://fungi.ensembl.org/Ustilago_maydis/Info/Index.

We further investigated the known upstream regulators of the TOR complex in the pathogenic fungal lineages (Table 1, Table S1). FKBP12 is reported to mediate the inhibitory effects of RAP on TOR by targeting the FRB domain (Heitman et al., 1991; Michnick et al., 1991). Orthologues of FKBP12 are found in all the investigated fungal genomes, suggesting the possible rapamycin‐sensitive properties in these fungal kingdoms. In animals, the TSC1 (tuberous sclerosis complex 1)‐TSC2 (tuberous sclerosis complex 2) heterodimer acts as a key regulator of TOR signalling. The TSC1‐TSC2 functions as a GTPase activating protein (GAP) for the small GTPase Rheb (Ras homologue enhanced in the brain) (Huang & Manning, 2008; Nakashima & Tamanoi, 2010). After activation, Rheb binds to the TORC1 complex and enhances its activity. We find that TSC1‐TSC2 only exists in *B. graminis* f. sp. *tritici*, *M. oryzae*, and *U. maydis*; however, Rheb homologues are identified in all fungal species in the present study, suggesting the altered TSC1/TSC2‐Rheb pathway in these species. Phospholipase D1 (PLD1) is involved in the Rheb action towards mTORC1. PLD1 has been reported to be indispensable for the class III phosphatidylinositol 3‐kinase VPS34‐mediated activation of TOR (Yoon et al., 2011). The VPS34‐PLD1 pathway has been identified in all the investigated fungal kingdoms. The AMP‐activated protein kinase (AMPK)‐TSC2 signalling pathway is another evolutionary conserved regulatory element of the TOR pathway (Inoki et al., 2006). Potential homologues of AMPK are conserved throughout the species examined. The phosphoinositide‐dependent kinase 1 (PDK1), phosphatase and tensin homologue (PTEN), and protein kinase B (PKB) (also known as AKT) proteins are another pathway that is wired to TOR through the TSC proteins. Homologues of PKB are conserved in the examined species. With the exception of *V. dahliae*, we find homologues of PDK and PTEN in all the species. The conserved guanine nucleotide exchange factor VAM6 can control TORC1 activity by activating the EGO complex, which consists of the Rag GTPase GTR1 and GTR2 along with EGO1 and EGO3 (Valbuena et al., 2012). We find presumptive VAM6 orthologues in the species in this study except for *F. oxysporum*, *P. infestans*, and *P. striiformis* f. sp. *tritici*. GTR1/GTR2 homologues are identified in the investigated pathogens; however, EGO1 and EGO3 are unique to *S. cerevisiae* and not identified in the other species, indicating that the EGO complex can be formed only in *S. cerevisiae*. We also examined the other well‐characterized upstream regulators of the TOR complex including GATOR1/2 and TTT‐R2TP (Bar‐Peled et al., 2013; David‐Morrison et al., 2016; Kim et al., 2013; Shen et al., 2018). These proteins are conserved throughout the organisms except in *U. maydis*.

We also identified several downstream effectors targeted by the TOR signalling (Tables 1 and S1). The AGC kinase SCH9, a homologue of the p70 S6 kinase (S6K), is the best‐characterized substrate of TORC1 (Deprez et al., 2018). SCH9/S6K and its substrate ribosome protein‐small subunit6 (RPS6) are involved in the TOR regulation of mRNA translation (Meyuhas, 2008). Homologues of SCH9 are identified in all the species studied through a BLASTp search. In *B. graminis* f. sp. *tritici*, an SCH9 homologue is identified, though RPS6 is not (Table 1, Table S1). This situation may indicate rewriting of this pathway in *B. graminis* f. sp. *tritici*. In both yeast and mammals, autophagy is an evolutionarily conserved cellular degradation pathway modulated by TOR; it controls the degradation of cytoplasmic contents and is hierarchically regulated by autophagy‐related genes (ATGs) (Cai et al., 2022; Mizushima, 2010). ATGs are identified in all the species investigated, indicating the conserved role of autophagy in the fungal kingdom. TORC1 regulates ribosome biogenesis through directly phosphorylating the split Zn‐finger transcription factor SFP1. Deletion of SFP1 induces reduced ribosome biogenesis and eventually results in a dramatic increase in TORC1 activity (Lempiäinen et al., 2009; Tai et al., 2023). Potential homologues of SFP1 are identified in most species investigated, with the exceptions of *B. graminis* f. sp. *tritici*, *F. graminearum*, *P. infestans*, and *U. maydis*. The PP2A phosphatase SIT4 is another proximal effector targeted by the TORC1 complex. SIT4 could interact with the TOR‐associated protein 42 (TAP42) in a TORC1‐dependent manner (Rohde et al., 2008). SIT4 and TAP42 regulate the transcriptional response of several TORC1‐controlled genes to glucose and the developmental cycle (Alfatah et al., 2021; Shertz et al., 2010). In *P. infestans*, a homologue of SIT4 is identified and a TAP42 homologue is not. The AGC‐family kinase 2 (YPK2) functions as a direct substrate of TORC2 and is involved in ceramide synthesis (Aronova et al., 2008; Shertz et al., 2010). Potential homologues of YPK2 are identified in all the species studied.

In conclusion, the two TOR complexes and a large part of the TOR signalling pathway molecular components are well conserved among the fungal species examined. In some cases, the homologues of some components are absent, and this result may mean a failure to identify them with the BLASTp method because of the insufficient similarity. In addition, these situations may suggest rewriting the pathway.

## 4 TOR SIGNALLING PATHWAY IS INVOLVED IN THE PATHOGENESIS OF FUNGI

**FIGURE 2:**
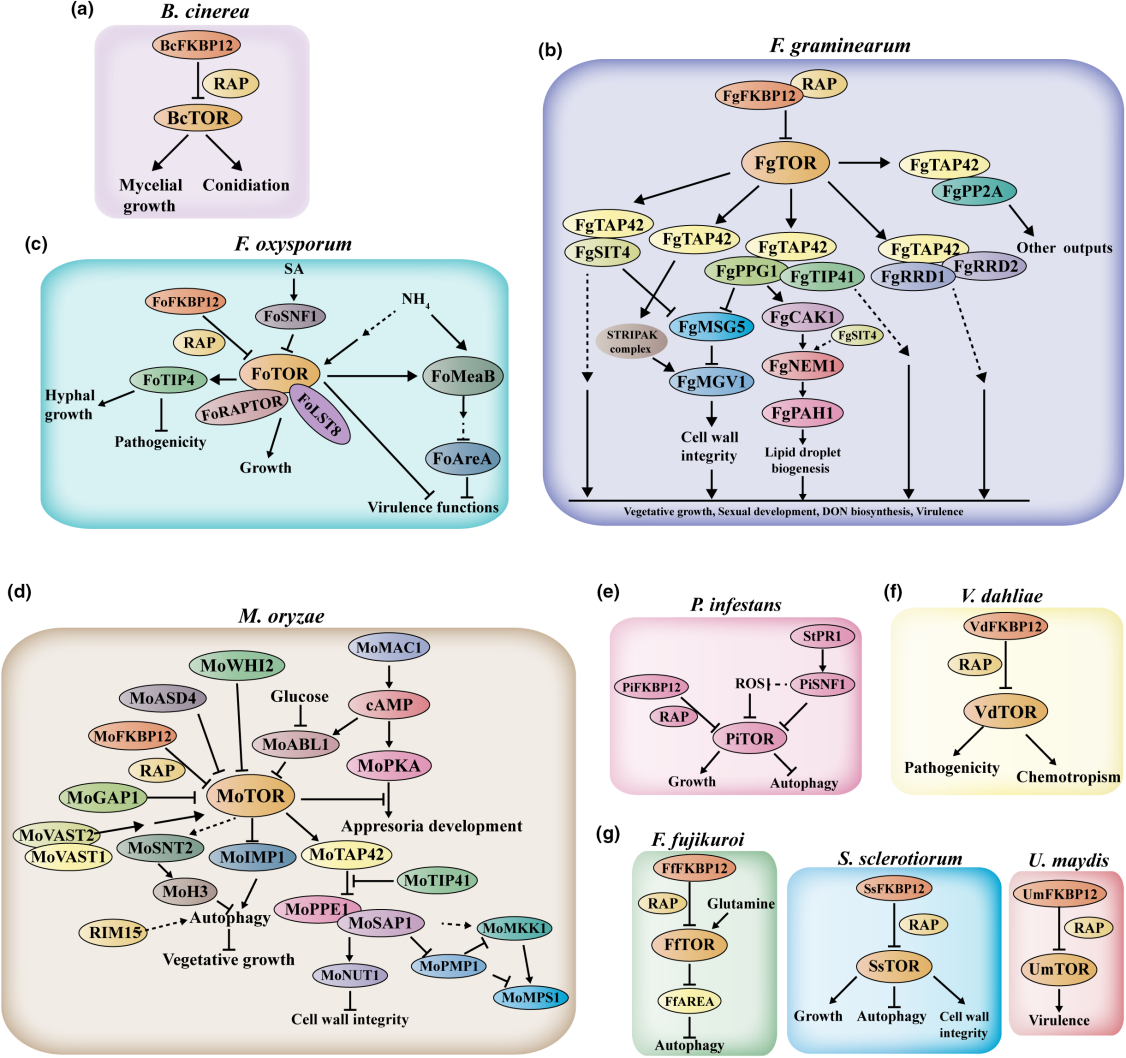
TOR signalling pathway governs key processes that strikingly affect fungal pathogenesis. Components of the TOR pathway in *Botrytis cinerea* (a), *Fusarium graminearum* (b), *Fusarium oxysporum* (c), *Magnaporthe oryzae* (d), *Phytophthora infestans* (e), *Verticillium dahliae* (f), and other representative plant‐pathogenic fungi (g) are shown. ABL1, carbon‐responsive gene; AreA, global nitrogen regulator; ASD4, the GATA transcription factor‐encoding gene; CAK1, protein kinase; cAMP, monobutyryl cyclic AMP; CaMV, cauliflower mosaic virus; FKBP12, FK506 binding protein 12; GAP1, amino acid permease; IMP1, vacuolar protein required for membrane trafficking; LST8, lethal with SEC. 13 protein 8; MAC1, the adenylate cyclase; MeaB, bZIP protein; MGV1, mitogen‐activated protein kinase gene 1; MKK1, mitogen‐activated protein kinase (MAPK) kinase; MSG5, phosphatase similar to yeast Msg5; NEM, protein phosphatase; NUT1, a major nitrogen regulatory gene; PAH, phosphatidate phosphatase; PKA, protein kinase A; PMP1, a tyrosine‐protein phosphatase; PP2A, protein phosphatase 2A; PPE1, homologue to *Saccharomyces cerevisiae* Sit4/Ppe1; PR1, pathogenesis‐related protein 1; *R. solanacearum*, *Ralstonia solanacearum*; RAP, rapamycin; RAPTOR, regulatory‐associated protein of mTOR; ROS, reactive oxygen species; RRD, resistance to rapamycin deletion 2; SIT4, PP2A phosphatase; SNF1, sucrose non‐fermenting 1; SNT2, named for the presence of the DNA‐binding domain SaNT; STRIPAK, striatin‐interacting phosphatases and kinases; TAP42, Tor associated protein 42; TIP41, Tap42‐interacting protein 41; TOR, target of rapamycin; VAST, VASt domain‐containing protein; WHI2, a homologue of *S. cerevisiae* Whi2 (Whisky2); *X. citri, Xanthomonas citri*.

Numerous studies have revealed that TOR signalling participates in the growth and pathogenicity of fungi. Moreover, environmental conditions, especially nutrients, could control TOR activity and then modulate the virulence programme in the fungi. In this section, we review the known progress made in the TOR pathway in various fungi (Figure 2).

### 4.1 Botrytis cinerea

The necrotrophic phytopathogenic fungus *B. cinerea* is a widespread plant pathogen and can infect numerous important economic and horticultural crops. This genus leads to severe pre‐ and post‐harvest grey mould rot or Botrytis blight and causes enormous economic losses worldwide (Bi et al., 2023; Chen, Zhang, et al., 2023). The *B. cinerea* genome contains a single orthologue of FKBP12, and *Bc*FKBP12 could mediate rapamycin sensitivity in *B. cinerea*, indicating that *Bc*FKBP12 ensures FKBP12 typical functions conserved in other species (Meléndez et al., 2009). Intriguingly, the *Bc*FKBP12 deletion in *B. cinerea* strain B05.10 did not affect its pathogenic development, while it was reported to cause a reduction of the virulence of strain T4 (Gioti et al., 2006; Meléndez et al., 2009). The involvement of *Bc*FKBP12 in host colonization may diverge between different strains. Recently, scientists have further characterized the presence of conserved TOR signalling of *B. cinerea* and its role in the regulation of growth and pathogenicity (Xiong et al., 2019). Moreover, pharmacological assays and bioinformatics analysis of RNA‐seq data showed that rapamycin exhibited a strong inhibitory effect on *B. cinerea* vegetative growth and sporulation. *BcTOR* silencing by plant‐mediated RNAi could efficiently reduce the pathogenicity of *B. cinerea* to *A. thaliana*, potatoes, and tomatoes.

### 4.2 Fusarium graminearum

*Fusarium graminearum*, a cosmopolitan fungal pathogen, causes Fusarium head blight (FHB) disease on various small‐grain cereals. *F. graminearum* not only reduces the yield production of infested grains but also produces mycotoxins including deoxynivalenol (DON) and zearalenone in crops, which are hazardous to animals and humans (Chen et al., 2019; McMullen et al., 1997; Pestka & Smolinski, 2005). Yu et al. (2014) first identified and characterized nine pivotal components of the TOR signalling pathway in *F. graminearum* and further investigated their biological, genetic, and biochemical functions. Rapamycin exhibits a strong inhibition of mycelial growth, hyphal branching, and septum formation of *F. graminearum*. Collectively, they defined the regulatory framework of the TOR pathway in regulating the virulence and vegetative differentiation of *F. graminearum*. They demonstrated that *Fg*SIT4, *Fg*PPG1, and *Fg*TIP41, three key components of the TOR signalling pathway, are involved in regulating conidiation and DON biosynthesis. No detectable DON can be found in the Δ*FgSIT4* mutants, and the amount of DON produced by Δ*FgPPG1* mutants showed no difference from that produced by the wild‐type strain. The amount of DON produced by Δ*FgTIP41* showed a significant decline compared with the wild‐type strain. Since then, extensive studies about the *F. graminearum* TOR pathway have been carried out. Antofine is a phenanthroindolizidine alkaloid and exhibits activities that dampen the growth of a variety of microorganisms (Mogg et al., 2019). However, the direct targets of antofine have not been identified. The TOR signalling pathway component *Fg*RRD2 (resistance to rapamycin deletion 2) is a target of antofine (González & Hall, 2017; Mogg et al., 2019). The plant‐derived compound antofine could be another inhibitor to dissect the TOR signalling function and may be used as a biofungicide to control plant fungal disease. Lipid droplets (LDs) are cellular organelles that control lipid metabolism (Wilfling et al., 2014). Rapamycin‐mediated *Fg*TOR inhibition rapidly promoted LD biogenesis through the *Fg*PPG1/SIT4 signalling branch (Liu et al., 2019). *Fg*PPG1 affected the downstream *Fg*NEM1/SPO7‐*Fg*PAH1 (phosphatidate phosphatase) phosphatase cascade and eventually regulated fungal development and virulence in *F. graminearum*. Furthermore, LD biogenesis induced by rapamycin was also observed in various filamentous fungi (Liu et al., 2019). In conclusion, LD biogenesis is a widely conserved downstream target of the TOR signalling pathway. Pathogens including *F. graminearum* could modulate their growth and metabolism to respond to environmental cues. They also found that the amount of the mycotoxin DON in the Δ*FgPAH1* and Δ*FgNEM1* mutants was significantly decreased, further indicating the role of the TOR pathway in regulating the virulence of *F. graminearum*. Nitrogen sensing plays a role in *F. graminearum* pathogenesis in plants. Both the TOR and the glutamine synthetase (GS)‐dependent signalling pathways mediate nitrogen sensing in *F. graminearum*, and two 14‐3‐3 proteins *Fg*BMH1 and *Fg*BMH2 are involved in the above regulation, DON production and mycelial growth (Brauer et al., 2020). The molecular interconnection between 14‐3‐3s and TOR‐ or GS‐induced signalling should be further clarified in future work. The STRIPAK complex was first characterized in mammalian cells and controls a series of pivotal cellular processes (Shi et al., 2016). A recent study indicated that this complex governed the *F. graminearum* development and virulence through orchestrating cell wall integrity signalling (Chen, Liu, et al., 2023). Mutations that delete individual components of this complex were observed to cause a significant reduction in fungal vegetative growth and sexual development and dramatically attenuate virulence. A reduction in DON production was also observed in the STRIPAK mutants compared to the wild type. This complex could interact with the mitogen‐activated protein kinase Mgv1 to regulate cell wall integrity. Further experiments revealed that the TOR pathway was linked with the STRIPAK complex through the TAP42‐PP2A cascade. The connection between the TOR signalling pathway and the STRIPAK complex in this study also provides a framework for future research about the function of the TOR pathway in the cell wall integrity pathway.

### 4.3 Fusarium oxysporum

The hemibiotrophic fungus *F. oxysporum* is a soilborne pathogen and can infect a series of economically important crops including bananas, cotton, potatoes, and tomatoes (Dean et al., 2012). This pathogen causes plant root rot, wilting, and necrosis, resulting in massive yield loss and plant death (Geiser et al., 2013). Separate teams showed that RAP could dampen the hyphal growth and development of *F. oxysporum* in a dose‐dependent manner (Li et al., 2021; López‐Berges et al., 2010a). Furthermore, the second‐generation TOR inhibitors also inhibited the hyphal growth of *F. oxysporum* (Li et al., 2021). They also observed a synergistic effect when *F. oxysporum* was treated with a combination of RAP and Torin1, indicating that TOR inhibitors simultaneously target the TOR pathway in *F. oxysporum*. Previous research showed that nitrogen limitation was proposed to be a decisive signal to promote the expression of pathogen virulence genes (López‐Berges et al., 2010a, 2010b). The expression of the *TOR* genes changed when *F. oxysporum* was grown in a minimal liquid medium containing 25 mM NH_4_NO_3_ (López‐Berges et al., 2010a). In addition, they revealed that invasive hyphal growth of *F. oxysporum* was dependent on nitrogen source and promoted by RAP, indicating the involvement of TORC1 in regulating the infection‐related processes in *F. oxysporum*. The *F. oxysporum* mutant D122, which was disrupted in a BAH/PHD‐containing transcription factor SNT2, showed impaired pathogenicity in plants (Denisov et al., 2011). In the D122 mutant, the expression levels of some genes associated with the TOR kinase pathway exhibited a significant change compared with that in the wild‐type isolate. Among these genes, two genes related to the autophagy pathway, *IDI4* and *PDC*, were upregulated in the mutant D122 and increased autophagosome abundance in *F. oxysporum*. Thus, TOR kinase is reported to play an important role in *F. oxysporum* autophagy regulation. In conclusion, *SNT2* function and pathogen pathogenicity are connected to the TOR pathway and autophagosome abundance. In a recent study, scientists investigated a gene expression profile in *F. oxysporum* upon *Fo*TOR inhibition. They revealed that the TOR pathway was indispensable for numerous metabolic processes, including ribosome biogenesis and cell wall‐degrading enzymes (CWDEs) (Li et al., 2021). Furthermore, they identified a new component of the *Fo*TOR pathway, *Fo*TOR1 interacting protein 4 (*Fo*TIP4). *Fo*TIP4 regulated the hyphal growth and pathogenicity of *F. oxysporum* by binding to the promoters of ribosome biogenesis‐ and CWDE‐related genes. Their results provide new insights into the molecular mechanism of the function of the *F. oxysporum* TOR pathway and further increase the possibility of the TOR pathway as a promising target for controlling the Fusarium wilt caused by *F. oxysporum*. In plants, salicylic acid (SA) functions as a phytohormone that activates plant disease resistance system (Ding et al., 2018). SA can enter the *F. oxysporum* cells and efficiently arrest hyphal growth, sporular production, and pathogenicity of this pathogen (Li et al., 2022). This study further identified that SA fought against *F. oxysporum* by targeting the SNF1‐TORC1 pathway. The interference of *Fo*TOR1 through the host‐induced gene silencing (HIGS) method could potently block the occurrence of Fusarium wilt. The invasive growth in *F. oxysporum* is affected by environmental cues, for instance, pH and nutrient status (López‐Berges et al., 2010a). TORC1 could promote pathogen cell growth in response to nutrient sufficiency. Navarro‐Velasco et al. (2023) found that the uncontrolled activation of TORC1 signalling, through inactivation of the negative regulator TSC2 under nutrient‐limiting conditions, attenuated the pathogen *F. oxysporum* virulence, which was shown in the impaired cellophane penetration ability and vegetative hyphal fusion or invasive growth. This further indicates the relevance of the TORC1 signalling with pathogenicity in a fungal pathogen.

### 4.4 Magnaporthe oryzae

*Magnaporthe oryzae* is the causal agent of the devastating blast of cultivated rice (*Oryza sativa*) and causes severe yield and substantial economic losses worldwide. During infection, this pathogen forms specialized structures called appressoria to enter the host cells. There has been a lot of progress in understanding *M. oryzae* TOR signalling (Li et al., 2023; Marroquin‐Guzman et al., 2017; Marroquin‐Guzman & Wilson, 2015; Sun et al., 2018, 2019). TOR signalling pathway is a powerful inhibitor of appressorium formation (Marroquin‐Guzman & Wilson, 2015). A *M. oryzae* mutant strain Δ*asd4*, which lacked a functional copy of the GATA transcription factor‐encoding gene *ASD4*, could not form appressoria. In Δ*asd4* mutants, the TOR pathway was activated, and exposing these mutants to the TOR inhibitor RAP could restore appressorium development. TOR inhibition of appressorium formation was modulated by the intracellular glutamine levels and functioned downstream of the protein kinase A catalytic subunit (cPKA). This study underpins the regulatory mechanism of appressorium development, and the TOR function in this process could be leveraged to develop strategies against this pathogen. Soon after, the function of TOR signalling in *M. oryzae* was dissected (Marroquin‐Guzman et al., 2017); they found that the cellular glucose‐regulated TOR activity and a novel carbon‐responsive gene *ABL1* were responsible for this process. In the absence of glucose, ABL1 could inhibit TOR activity, which sheds new light on the inhibitory signals of the TOR pathway. They also elucidated that the novel glucose‐ABL1‐TOR signalling axis modulated cell cycle quiescence at G1/G0, and eventually controlled appressoria development. Furthermore, they elaborated on a novel molecular mechanism of *M. oryzae* infection and suggested that the TOR‐IMP1‐autophagy branch signalling dictated membrane homeostasis to regulate plant–microbe biotrophic interface longevity (Sun et al., 2018). They discovered a novel vacuolar protein IMP1 through screening *M. oryzae* mutant strains resistant to RAP and revealed that IMP1‐dependent autophagy ensured biotrophic interfacial membrane integrity. However, the precise molecular mechanism of autophagy during biotrophic growth remained unclear until they provided new molecular insights into *M. oryzae* appressorium morphogenesis (Sun et al., 2019). Appressorial morphogenesis requires activated cAMP/PKA signalling (downstream of cPKA) and inactivated TOR signalling (TOR_off_), and TOR_off_ arrested mitosis in the G2 phase, which induced autophagy (Marroquin‐Guzman et al., 2017; Marroquin‐Guzman & Wilson, 2015). They investigated how cAMP/PKA signalling is connected to cell cycle progression and autophagy. They addressed a feed‐forward adenylate cyclase MAC1‐cAMP‐TOR‐adenylate cyclase subnetwork that could reinforce cAMP/PKA‐dependent appressorium formation in response to favourable environmental conditions (Sun et al., 2019). Recently, it has been found that the *M. oryzae* serine/threonine protein kinase RIM15 mediates invasive hyphae growth through regulating cycles of autophagy and glutaminolysis (Li et al., 2023). RIM15 deletion mutant strains showed attenuated extensive biotrophic growth and suppressed plant defence, indicating that RIM15 was required for biotrophic growth. RIM15‐mediated phosphorylation of glutamate dehydrogenase activated the TOR signalling pathway and eventually inhibited autophagy. The work provided molecular and metabolic evidence of fungal biotrophy in host cells and could open new avenues for pathogen weaknesses.

Other scientists have also contributed to the research of TOR signalling in *M. oryzae*. In plant pathogens, cell wall integrity (CWI) is indispensable for appressorium function and pathogenicity. The mitogen‐activated protein kinase (MAPK) kinase *Mo*MKK1 played a pivotal role in the CWI pathway. Qian et al. (2018) characterized that *Mo*MKK1 interacted with *Mo*PPE1, which is a homologue of serine/threonine protein phosphatase SIT4/PPE1 and is required for the growth and pathogenicity of *M. oryzae*. *Mo*PPE1 interacted with *Mo*SAP1 and functioned as an adaptor complex to coordinate CWI and TOR signalling. *Mo*PPE1 coordinated the TOR pathway by interacting with *Mo*TAP42. Activated TOR signalling showed a suppression effect on the CWI pathway, which eventually resulted in impairment in appressorium function and pathogenicity. This study revealed the new *Mo*MKK1–*Mo*PPE1–*Mo*SAP1 signalling axis, promoted the understanding of pathways related to pathogenicity, and provided novel targets to control the rice blast. Soon after, they uncovered that the TAP42‐interacting protein 41 (*Mo*TIP41) interacted with *Mo*PPE1 and thus participated in the TOR signalling pathway to modulate the CWI pathway and the pathogenicity of *M. oryzae* (Qian et al., 2021). In *S. cerevisiae*, TIP41 interacts with and inhibits TAP42, which activates PPE1 and dampens the TOR pathway (Jacinto et al., 2001), whereas *Mo*TIP41 could not interact with *Mo*TAP42, indicating that the function of components of the TOR signalling pathway may be diverse in different species. Emerging evidence shows that epigenetic regulators of histone proteins play vital roles in the growth and virulence of plant fungal pathogens. He et al. (2018) demonstrated that *Mo*SNT2, a homologue of yeast SNT2, is involved in autophagy, autophagy‐dependent pathogenicity, CWI, and oxidative stress response. They revealed that *Mo*SNT2 mediated epigenetic control of gene expression through binding the acetylated H3 histone and recruiting histone deacetylase. Moreover, they observed that the expression level of *MoSNT2* was dependent on *Mo*TOR, and *Mo*SNT2‐mediated infection‐associated autophagy and autophagic pathogenic growth through coordination with the *Mo*TOR kinase pathway. However, the precise molecular mechanism between *Mo*TOR and *Mo*SNT2 was undissected. In conclusion, this study offered a novel histone‐based avenue for antifungal discovery. Shi et al. (2021) investigated that the homologue of yeast WHI2 (*Mo*WHI2) interacted with the putative phosphatase *Mo*PSR1 and regulated appressorium formation and pathogenicity in *M. oryzae*. The regulation of appressorium formation mediated by *Mo*WHI2 and *Mo*PSR1 was closely related to the cAMP levels and the *Mo*TOR signalling, indicating the conserved and pivotal role of the *Mo*TOR pathway in fungal appressorium formation and pathogenicity. In yeast, the general amino acid permease 1 (GAP1) is an amino acid transporter, mediates the amino acid signalling pathway, and modulates the activity of TORC1 kinase complex to sustain cell growth and mitosis (Bar‐Peled & Sabatini, 2014). In *M. oryzae*, *Mo*GAP1 functioned upstream of the TOR signalling and regulated the autophagy process (Huang et al., 2022). *Mo*GAP1 deletion affected the conidial development, altered sensitivity to RAP, and increased the level of autophagy in *M. oryzae*. Moreover, *Mo*GAP1 was also involved in cell wall stress and carbon source stress. This conclusion further confirmed the involvement of the TOR signalling pathway in the pathogenicity of *M. oryzae*. However, the role and mechanism of *Mo*GAP1 in the infection of *M. oryzae* need further exploration. Zhu et al. (2021, 2023) revealed that *Mo*VAST2 (VAST domain‐containing protein 2) interacted with and colocalized with *Mo*ATG8 and *Mo*VAST1 (VAST domain‐containing protein 1), which was previously reported to be an autophagy regulator. They found that *Mo*VAST2 deletion mutant strains showed high sterol accumulation, inappropriate autophagy progress, low sphingolipids, and the compromised activity of the TOR complexes. They confirmed that the combination of *Mo*VAST1 and *Mo*VAST2 regulated the activity of the TOR pathway and kept the balance of lipid homeostasis and autophagy in *M. oryzae* (Zhu et al., 2023). Their findings further demonstrated that the TOR pathway was involved in the vegetative growth and pathogenicity of phytopathogenic fungi.

### 4.5 Phytophthora infestans

*Phytophthora infestans* causes late blight of potato (*Solanum tuberosum*) and tomato (*Solanum lycopersicum*) and incurs enormous quantitative and qualitative losses on agriculture (Fry, 2016; Haas et al., 2009; Wang & Long, 2023). The Great Irish Famine that began in the mid‐1840s was caused by the potato late blight and led to a devastating impact on people (Geber et al., 2019). Bioinformatics analysis of the *P. infestans* kinome identified the existence of two homologues of mTOR protein (Judelson & Ah‐Fong, 2010). Recently, researchers analysed the key components of the TOR signalling pathway in detail and dissected that the TOR pathway was conserved in *P. infestans* (Zhang et al., 2021). A series of TOR‐specific inhibitors, for instance, RAP, AZD, KU, and Torin1, was exposed separately or co‐applied to this pathogen and significantly inhibited the growth and virulence of *P. infestans*, which was further verified by transcriptome data. The detailed analysis of the transcriptome data indicated that the *Pi*TOR signalling pathway participated in protein biosynthesis, which was consistent with the function of the TOR pathway in other species. In their study, the co‐application of RAP and AZD showed higher inhibition efficacy than that through individual use, which provides alternative methods for controlling late blight by using TOR‐targeted drugs. Moderate levels of reactive oxygen species (ROS) in pathogens are necessary for vegetative growth and pathogenicity. Luo et al. (2021) investigated how *P. infestans* responded to excessive H_2_O_2_ induced by the host immune or the environment. They revealed that the exogenous addition of H_2_O_2_ inhibited vegetative growth and reduced the pathogenicity of *P. infestans*, which mimicked that of pathogens treated with RAP. The analysis of the transcriptome, proteome, and phosphorylation omics revealed that the TOR signalling pathway was involved in *P. infestans*' response to ROS stress through operating the *Pi*TOR2‐mediated autophagy pathway, suggesting the conserved role of autophagy in ROS stress response. Furthermore, they performed the *Pi*TOR silencing through host‐induced gene silencing (HIGS), which enhanced plant resistance to *P. infestans*. Further research is needed to verify the precise molecular mechanism of TOR‐mediated autophagy in ROS stress response. Recently, they identified the corresponding genes of the TOR pathway in *P. infestans* and found that the *Pi*SNF1 and *Pi*AMPK kinase complex was targeted by *St*PR1 (*S. tuberosum* pathogenesis‐related protein 1), which played a vital role in plant defence against *P. infestans* (Luo et al., 2023). *St*PR1 changed the AMPK‐mediated phosphorylation to downstream target proteins, for instance, the acetyl‐CoA carboxylase (ACC) protein, and the inhibited AMPK activity repressed the vegetative development and pathogenicity of *P. infestans*. Furthermore, they revealed that *St*PR1–*Pi*AMPK interaction disrupted ROS homeostasis. In conclusion, *St*PR1 suppressed *P. infestans* by targeting the AMPK kinase complex. The AMPK–TOR complex may be another potential target for controlling plant disease in crops.

### 4.6 Verticillium dahliae

*Verticillium dahliae* is a notorious and widespread soilborne fungal pathogen and can infect a broad range of dicotyledonous plants, including cotton, eggplant, potato, and tomato, causing tremendous losses annually (Chen et al., 2021; Fradin & Thomma, 2006; Inderbitzin & Subbarao, 2014). Verticillium wilt caused by *V. dahliae* is a cancer of cotton crops and is hard to control because of the longevity of *V. dahliae* in soil as its resting structures are microsclerotia. Progress in the TOR signalling in *V. dahliae* is limited. Li et al. (2019) found that RAP retarded the *V. dahliae* mycelial growth and conidial development and revealed the existence of the conserved TOR pathway. Furthermore, they performed the RNA‐seq experiments under TOR inhibition. They revealed that the differentially expressed genes (DEGs) were mainly involved in the regulation of cell growth and invasion, for instance, cell wall‐degrading enzymes (CWDEs). Their results together supported the conclusion that the reduced *Vd*TOR activity influenced the mycelial growth and pathogenicity of *V. dahliae*. Through dissecting the function of the TOR pathway, we conclude that *Vd*TOR is a potential target to control Verticillium wilt. Pathogens can sense the resources in the environment such as nutrients, toxic substances, and other individuals and adjust their hyphae growth to survive accordingly (Brand & Gow, 2009; Leeder et al., 2011). Vangalis et al. (2023) revealed that two components of the TOR pathway, GTR1 and TSC2, played a vital role in controlling *V. dahliae* chemotropism towards nutrients and plant signals. Both knockout mutants displayed abnormal fungal morphology and hyphal growth compared with wild‐type strains. The mutants both displayed increased chemotropic responses to diverse environmental stimuli. In addition, they found that GTR1 and TSC2 contributed to the virulence of *V. dahliae*, and autophagy dysfunction caused by TOR activity regulation accounted for the pathogenicity defect. Moreover, MAPK signalling was also involved in controlling *V. dahliae* chemotropism. Previous studies have implicated the interaction between TOR and MAPK pathway (Madrid et al., 2016). It is necessary to examine whether and how their crosstalk is involved in regulating *V. dahliae* chemotropism and pathogenicity.

### 4.7 Other representative plant‐pathogenic fungi

*Fusarium fujikuroi* is a prevalent pathogenic fungus and causes rice bakanae disease, which leads to enormous crop losses (Cen et al., 2020). *F. fujikuroi* is also known as the gibberellin producer and several other secondary metabolites, for instance, the red pigment bikaverin (Linnemannstöns et al., 2002). The biosynthesis of these secondary metabolites is controlled by the AreA‐ and glutamine synthetase (GS)‐mediated nitrogen regulation network (Linnemannstöns et al., 2002; Mihlan et al., 2003). Teichert et al. (2006) unravelled that the *Ff*TOR kinase was involved in the AreA‐mediated nitrogen regulation related to GA and bikaverin biosynthesis. They demonstrated that pathogens under the TOR kinase inhibition through exposure to RAP exhibited drastic growth defects. The *tor* knockout led to a lethal effect in *F. fujikuroi*. RAP significantly altered the gene expression level of AreA‐dependent genes, and the regulation was relevant to the nitrogen source and concentration. Furthermore, microarray analysis indicated that the TOR kinase affected the expression levels of genes related to translation control, ribosome biogenesis, carbon metabolism, and autophagy. For the first time, they showed the function of TOR protein in *F. fujikuroi*.

*Sclerotinia sclerotiorum* is a damaging phytopathogenic fungus that causes Sclerotinia stem rot in various crops worldwide (Bolton et al., 2006; Xu et al., 2018). It can survive for a long time in soil, which makes it difficult to control. It is necessary to develop new potential targets for managing this disease. Jiao et al. (2023) identified the conserved TOR signalling pathway in *S. sclerotiorum*. They revealed that silencing *Ss*TOR retarded the hyphal growth and the formation of sclerotia and compound appressoria of *S. sclerotiorum*. They further found that the TOR pathway was involved in various life processes in *S. sclerotiorum*, including abiotic stress response, CWI (through regulating the phosphorylation of a key MAP kinase *Ss*SMK3), and the autophagy pathway (via *Ss*ATG1 and *Ss*ATG13). Their study suggested that the *Ss*TOR pathway was a master regulator of the development and pathogenicity and could provide new routes for managing Sclerotinia stem rot.

The biotrophic phytopathogenic fungus *Ustilago maydis* infects most of the grasses including barley, maize, wheat, and sugar cane (Brefort et al., 2009; Djamei, 2023). The ectopic activation of TOR on *U. maydis* virulence has been dissected (de la Torre & Pérez‐Martín, 2022). The TOR pathway is present in *U. maydis*. They failed to obtain a null mutant in *tor1*, indicating that TOR was vital in *U. maydis*. The TOR pathway also played a conserved role in *U. maydis*: promoting ribosomal synthesis and repressing autophagy. The Rheb GTPase protein functions as a conserved TOR kinase regulator, is controlled by the TSC complex, and can activate TORC1 (Yang et al., 2021). They constructed gain‐of‐function mutants of the Rheb GTPase protein, which maintained the high activity of the TORC1 pathway. The Rheb‐activating mutations showed impaired infection ability. Meanwhile, they observed that the mutants were insensitive to pheromone signalling, which was TORC1‐independent. In their study, they observed that TORC1 was not involved in the negative regulation of virulence in *U. maydis*, which is contrary to studies in other phytopathogenic fungi (López‐Berges et al., 2010a; Yu et al., 2014).

In conclusion, it appears that the FKBP12‐TOR pathway is conserved in all the investigated fungi. Furthermore, the TAP42 and TIP4 proteins indeed govern various fungi development and virulence, which offers potential and promising targets to engineer broad‐spectrum resistance to various pathogens.

## 5 CROSSTALK BETWEEN PATHOGENS AND THE PLANT TOR SIGNALLING PATHWAY

**FIGURE 3:**
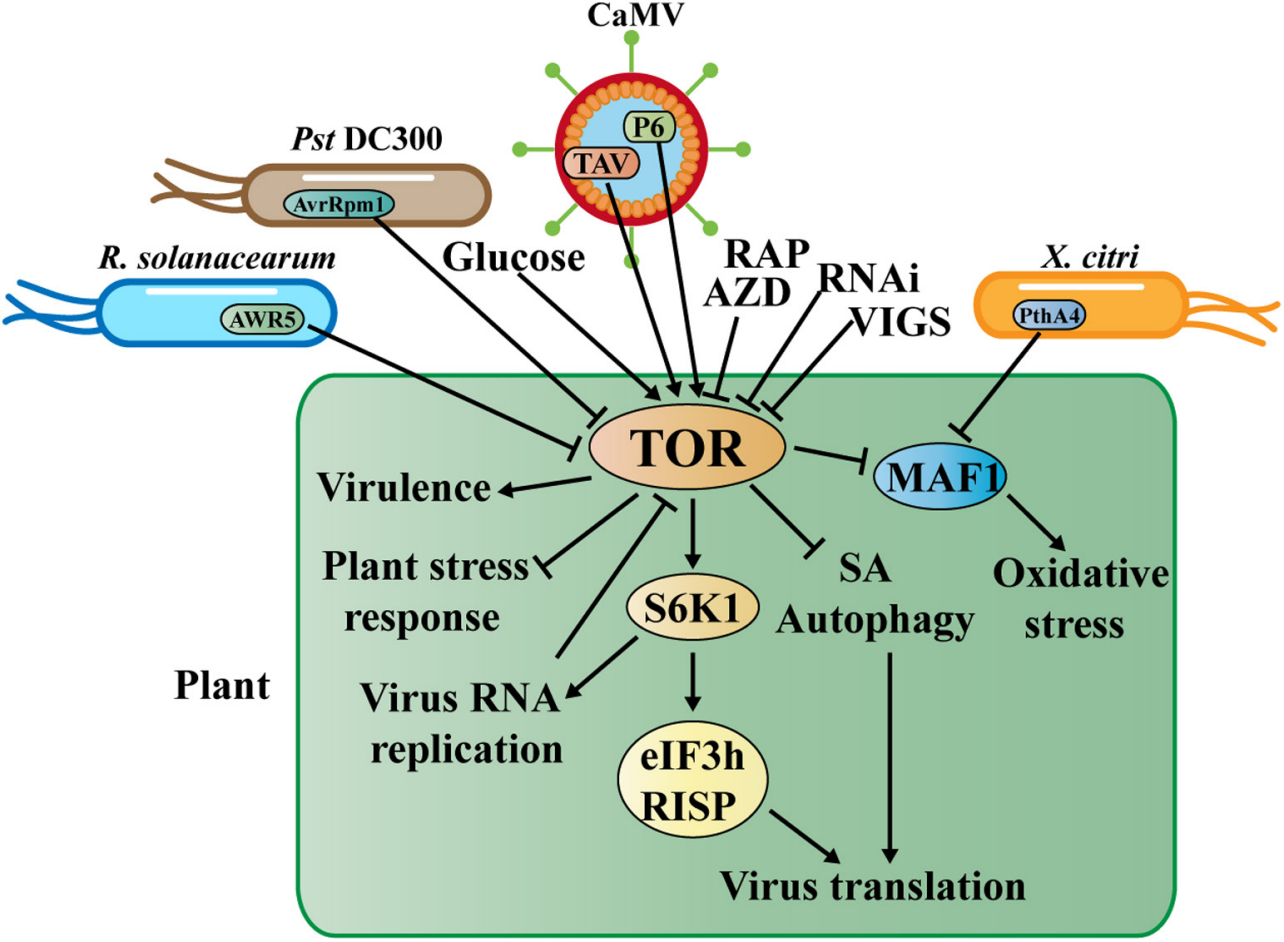
Pathogens affect the plant TOR signalling to drive their infection. Pathogens can recruit plant TOR/S6K1 signalling to facilitate their growth. TOR inhibition through inhibitor treatments, RNAi, or VIGS technologies primes immunity and pathogen resistance in plants. CaMV, cauliflower mosaic virus; Pst, *Pseudomonas syringae* pv. *tomato*; *R. solanacearum*, *Ralstonia solanacearum*; *X. citri*, *Xanthomonas citri*; TOR, target of rapamycin; S6K1, ribosomal protein S6 kinase; TAV, transactivator–viroplasmin; RNAi, RNA interference; RISP, reinitiation‐supporting protein; AWR5, type III effector; SA, salicylic acid; P6, a versatile viral effector; PthA4, a transcription activator‐like (TAL) effector; AvrRpm1, a *Pseudomonas* effector; VIGS, virus‐induced gene silencing.

In numerous studies, the plant TOR pathway is inferred as a vital switch from growth to defence to pathogens (Henriques et al., 2022; Margalha et al., 2019). Understanding the mechanism of the crosstalk between plants' TOR signalling pathways and pathogens will open new avenues for antifungal research (Figure 3). Various *Arabidopsis* mutants were impaired in TOR signalling, and plants treated with TOR inhibitor PP242 exhibited enhanced resistance to *F. graminearum* infection. Their results further confirmed the role of plant TOR in orchestrating growth–defence trade‐offs. Impaired TOR signalling in diverse TOR‐complex mutants or through TOR inhibitor treatment can confer resistance to the *F. graminearum* in the model plant *A. thaliana* (Aznar et al., 2018). Consistently, TOR inhibition by exposure to TOR inhibitor or using the virus‐induced gene silencing (VIGS) system increased tomato immune responses and disease resistance to *B. cinerea* (Marash et al., 2022). These studies made the TOR pathway another toehold in the plant response to recalcitrant fungal pathogens. The underlying mechanism of TOR–*Podosphaera xanthii* infection has been investigated (Chen, Qu, et al., 2023). TOR played a negative role in regulating cucumber resistance to pathogens. A phosphoproteomics analysis of *Cucumis* against *P. xanthii* attack under TOR inhibition revealed a series of candidate TOR substrates including proteins involved in plant hormone signalling, MAPK cascade signalling, and defence response‐related proteins, which provided rich resources to dissect TOR function in plant response to the pathogen.

The decreased plant TOR signalling pathway activity could enhance fungal pathogens infection, and the results were consistent with those in bacteria and viruses. In *A. thaliana*, TOR‐deficient RNAi‐silenced plants showed more resistance to cauliflower mosaic virus (CaMV) (Schepetilnikov et al., 2011). The CaMV viral re‐initiation factor transactivator/viroplasmin (TAV) could interact with the HEAT repeat domains of TOR1 and hijack TOR/S6K1 signalling to drive their replication. When challenged with watermelon mosaic virus (WMV), the TOR‐downregulated RNAi *Arabidopsis* lines showed less severe symptoms compared with the wild type, and AZD‐8055 application reduced WMV accumulation (Ouibrahim et al., 2015). Moreover, the CaMV viral effector P6 bound and activated the plant TOR kinase, thus suppressing SA‐dependent autophagy and eventually facilitating CaMV translation (Zvereva et al., 2016). The viral‐mediated suppression of the plant response predisposed plants to secondary infections with *Pseudomonas syringae* pv. *tomato* (Zvereva et al., 2016). Tomato bushy stunt virus (TBSV) uses and co‐opts host factors during replication (Nagy, 2016). TBSV replication partially depends on the yeast and plant TOR activity; retarded TOR activity reduced TBSV replication. The glycolytic pathway is one of the main targets of TORC1, and this pathway was also found to be critical for TBSV replication. Collectively, the TOR pathway was indispensable for efficient TBSV replication (Inaba & Nagy, 2018). *Ralstonia solanacearum* is the second most important bacterial plant pathogen and infects a series of important crops such as potato, tomato, eggplant, and some ornamentals. Recently, *R. solanacearum* has been widely used as an ideal model to study plant–pathogen interactions (Coll & Valls, 2013). It has been found that the *R. solanacearum* type III effector (T3E) AWR5 may directly or indirectly interact with the TOR pathway upstream PP2A regulatory or scaffold subunits and inhibit the activity of the TOR pathway (Popa et al., 2016). Additionally, heterologous overexpression of AWR5 in yeast induced massive transcriptomic changes that mimicked those caused by RAP. The results indicate that the plant TOR pathway is a bona fide target of plant pathogens. One study revealed that the *Pseudomonas* effector AvrRpm1 downregulated the translational level of *TOR*, and TOR downregulation contributed to defence against *P. syringae* and oomycete pathogen *Hyaloperonospora arabidopsidis* (Meteignier et al., 2017, 2018). MAF1 is an RNA polymerase III repressor and functions downstream of the TOR protein. TOR inhibition severely influences the phosphorylation levels of MAF1 (Ahn et al., 2019). The bacterial pathogen *Xanthomonas citri* effector PthA4 could interact with the sweet orange (*Citrus sinensis*) *Cs*MAF1 and repress Pol III transcription (Soprano et al., 2013, 2018). Future studies on the molecular mechanisms of TOR involved translational control in plants will help to understand how plants defend against pathogens. De Vleesschauwer et al. (2018) demonstrated the function of TOR signalling in rice. Their study indicated that TOR overexpression increased rice susceptibility to the bacterial leaf blight pathogen *Xanthomonas oryzae* pv. *oryzae* and the necrotrophic fungal pathogens *Cochliobolus miyabeanus* and *Rhizoctonia solani*; reduced TOR signalling showed enhanced resistance to these bacterial and fungal pathogens, which is in line with previous studies (Aznar et al., 2018; Meteignier et al., 2017; Ouibrahim et al., 2015). Recently, scientists found that a rice FKBP12 homologue functioned as a negative regulator in plant biotic stress response (Cheung et al., 2020). Ectopic overexpression of *OsFKBP12* in *Arabidopsis* reduced plant resistance towards the biotrophic pathogen *P*. *syringae* pv. *tomato*, and the *At*FKBP12‐knockout mutant enhanced the plant's ability to defend against this pathogen. However, whether the TOR pathway is involved in the *Os*FKBP12‐mediated plant stress response is not mentioned in the present study, which could be further validated in future research.

Hormones including SA and jasmonic acid (JA) are a central part of plant immune responses to plant pathogens, which is well established in various studies (De Vleesschauwer et al., 2014; Liu & Xiong, 2022; Pieterse et al., 2009). Independent studies report that TOR can subdue JA and SA signalling. TOR inhibition influenced JA content, and JA biosynthetic and signalling mutants displayed AZD8055‐resistant phenotypes (Song et al., 2017). In *Arabidopsis*, scientists detected the content changes of JA and its precursor in *raptor1b* mutants (Salem et al., 2017). In rice, the expression levels of genes related to SA and JA pathways and hormone contents significantly changed under TOR inhibition and in TOR and RAPTOR RNAi plants (De Vleesschauwer et al., 2018). Hence, the TOR pathway may modulate the SA and JA pathway to affect plant immunity. Further molecular evidence needs to be provided to understand whether and how the TOR pathway balances plant growth and defence response through hormones.

In conclusion, the above results in diverse plant species demonstrate that the activation of TOR can condition plant susceptibility to various pathogens. These results from different laboratories illustrate that the TOR pathway undeniably orchestrates plant growth–defence trade‐offs. However, the detailed molecular mechanism of TOR‐triggered plant resistance response alteration to pathogens is complicated. Scientists failed to detect enhanced susceptibility to *B. cinerea* in the *Arabidopsis* TOR overexpression plants, whereas TOR inhibition showed enhanced resistance to *B. cinerea* in the *Arabidopsis raptor1‐1* mutants and tomato (De Vleesschauwer et al., 2018; Marash et al., 2022). Moreover, some studies found that *Arabidopsis* TOR overexpression plants exhibited increased susceptibility to *P. syringae* pv. *tomato*; however, another study failed to detect enhanced susceptibility to this bacterium in their TOR overexpression lines (De Vleesschauwer et al., 2018; Meteignier et al., 2017). The divergences between different studies indicate that the role of the TOR pathway in regulating plant defence response may hinge on several factors, for instance, the different signal pathways affected by TOR activation and inhibition, the signal output strength, and the specialized interaction between various plants and pathosystems. Hence, more detailed experiments need to be performed to reveal the complex nuanced mechanisms.

## 6 TOR‐BASED THERAPIES TO COMBAT PATHOGEN INFECTION

**FIGURE 4:**
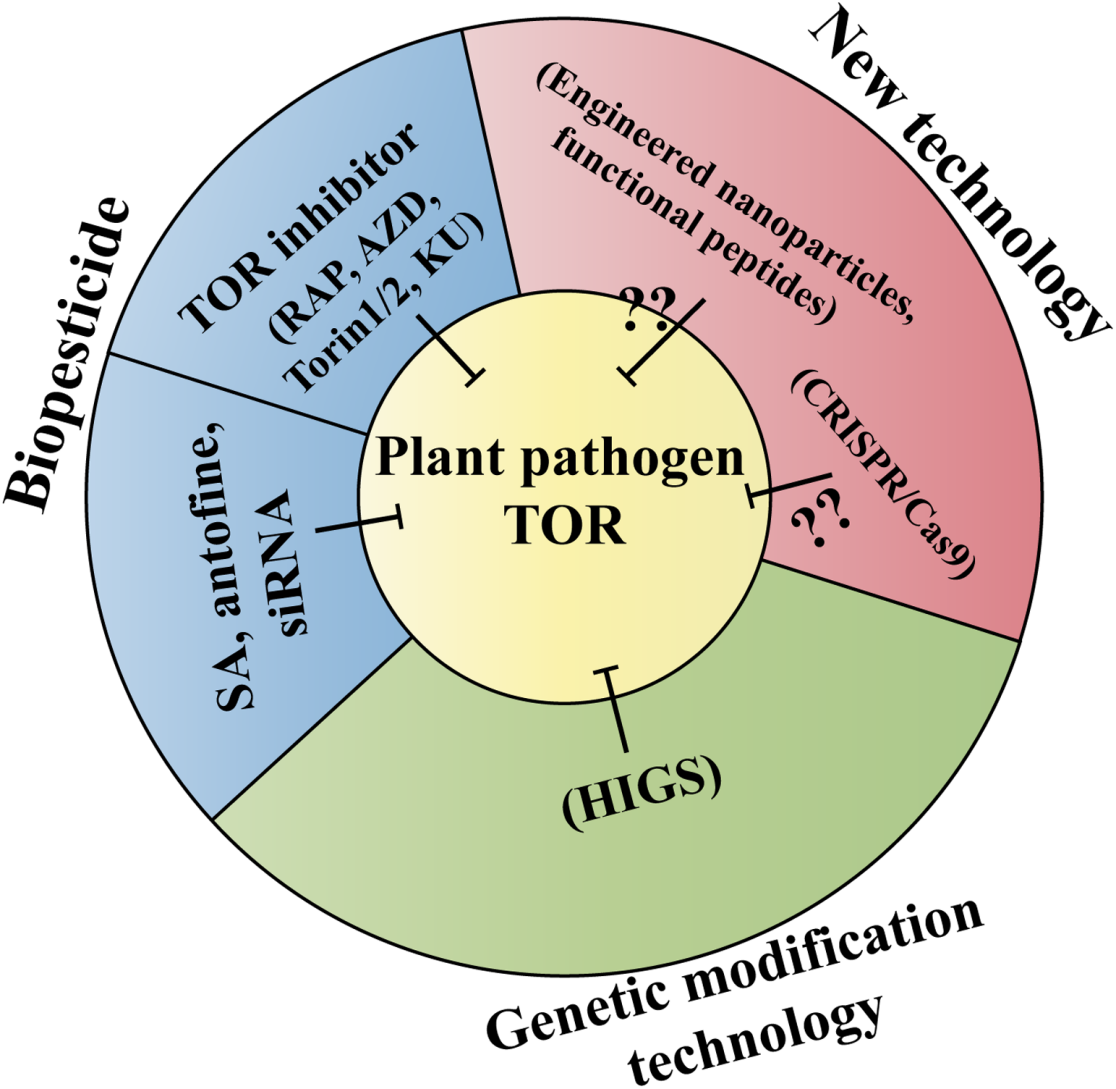
Schematic illustration of TOR‐based therapies for disease control. Genetic modification technology and biopesticide mentioned here are based on the literature survey conducted on the papers published recently. Whether and how some new technologies such as engineered nanoparticles or functional peptides can be applied to TOR‐based therapies needs further investigation.

Emerging evidence shows that the TOR pathway is a key pillar of growth and virulence in various plant pathogens and hence constitutes a vulnerable target for plant disease manipulation. As such, scientists have employed diverse strategies to manipulate the TOR pathway and combat plant pathogens. In this section, we discuss several examples of how scientists employ different mechanisms to exploit the TOR pathway to manipulate plant pathogens (Figure 4).

Chemical fungicides and biopesticides are global strategies used for crop protection nowadays. However, the use of fungicides and biopesticides results in nontarget environmental effects, bringing health risks and pollution, which threaten food safety and limit the application of conventional fungicides and biopesticides. Efforts that look for novel, efficacious, and environmentally sound plant‐protection products with low toxicity and high efficiency should be made in future plant protection. We find that the TOR‐specific inhibitors have begun to rise for plant disease control. The highly conserved structural organization of the TOR protein and the feature of the inhibitor‐sensitive TOR pathway in regulating the growth and virulence of the majority of pathogens enable the development of TOR inhibitor‐based strategies for antifungal therapies. A series of studies have indicated that rapamycin can dampen the fungal growth in various pathogens including *B. cinerea* (Meléndez et al., 2009; Xiong et al., 2019), *F. graminearum* (Brauer et al., 2020; Liu et al., 2019; Yu et al., 2014), *F. oxysporum* (Li et al., 2021; López‐Berges et al., 2010a), *M. oryzae* (Fernandez et al., 2014; He et al., 2018; Marroquin‐Guzman & Wilson, 2015), *P. infestans* (Luo et al., 2021; Zhang et al., 2021), *S. sclerotiorum* (Jiao et al., 2023), *U. maydis* (de la Torre & Pérez‐Martín, 2022), and *V. dahliae* (Li et al., 2019). Besides, the second‐generation TOR inhibitors, including AZD, Ku, and Torin1, are also widely applied to the growth inhibition in *F. oxysporum* (Li et al., 2021), *P. infestans* (Zhang et al., 2021), *U. maydis* (de la Torre & Pérez‐Martín, 2022), and *V. dahliae* (Li et al., 2019). Furthermore, the second‐generation TOR inhibitors show more drastic inhibitory effects compared with RAP in *P. infestans* and *U. maydis*. Meanwhile, scientists have revealed that the phytohormone SA functions as a biofungicide, enters the pathogen cells to subvert the TOR signalling pathway, and eventually fights against *F. oxysporum* (Li et al., 2022). The plant alkaloid antofine is a product produced by milkweed and exhibits growth inhibition activity in a variety of microorganisms (Mogg et al., 2019). Researchers find that antofine targets the TOR signalling pathway and inhibits the growth of *F. graminearum*, which provides a novel fungicide in agriculture. However, it should be noted that the TOR inhibitors can also inhibit the plant or human TOR pathway. We can control the dose of the selected TOR inhibitors to make them effective for fungal pathogens but pose no threat to plants or human health.

Among the conventional approaches to manipulating plant disease, host plant genetic resistance through genetic modification technologies appears to be the more durable and faster strategy (Kuo & Falk, 2020). Host‐induced gene silencing (HIGS) is an RNA interference (RNAi)‐based natural method used by plant hosts to transfer small RNAs (sRNAs) to defend themselves against invaders (Baulcombe, 2015). Transgenic HIGS is now being studied and discovered to be an effective method for plant disease control (Kuo & Falk, 2020). Several studies have reported that transgenic plants engineered to give stable HIGS targeting of the pathogen *TOR* gene can block the occurrence of plant disease. For instance, transgenic potato and tomato expressing double‐stranded RNA (dsRNA) of *BcTOR* through HIGS show enhanced resistance to *B. cinerea* by interfering with the expression of *BcTOR* (Xiong et al., 2019). Researchers engineered potato plants to produce sRNAs homologous to the mRNA for *PiTOR* genes (Luo et al., 2021). When they inoculated these transgenic potato lines with *P. infestans*, the transgenic lines showed increased resistance to late blight, indicating that the sRNA generated by the transgenic plants could be absorbed by *P. infestans* and induce the RNAi effect. It is reported that transgenic potato plants with interference of *FoTOR* genes also displayed a smaller lesion size than the wild type when exposed to *F. oxysporum*, and the mRNA level of *FoTOR1* was significantly decreased after inoculation (Li et al., 2022). They also revealed that exogenous application of the siRNAs of *FoTOR1* significantly inhibited the mycelial growth of *F. oxysporum*. Furthermore, the silencing of *SsTOR* through RNAi methods resulted in abnormal hyphal growth and sclerotia formation in *S. sclerotiorum*, indicating that *Ss*TOR could be a potential target of HIGS in constructing genetically modified resistant plants (Jiao et al., 2023). Taken together, the transgenic HIGS approach to target plant‐pathogenic fungal TOR has proved to be effective against several fungal types. However, off‐target effects must be considered when using the transgenic HIGS approach. Considering that TOR is a lethal gene in eukaryotes from plants to pathogens, we should make sure that the dsRNAs specifically target the pathogen TOR genes.

The strategies mentioned above directly target the pathogen's TOR protein. Recently, studies have revealed that different methods to manipulate the other key components of the TOR signalling pathway also play a role in mitigating the effect of the disease caused by phytopathogenic fungi. For example, important roles of the homologues of the Rag GTPase GTR1 and the GTPase‐activating protein TSC2 were identified in *V. dahliae* development and pathogenicity (Vangalis et al., 2023). The *Pi*SNF1 inhibition resulted in restricted vegetative growth and decreased pathogenicity in *P. infestans* (Luo et al., 2023). Therefore, these results expand the range of components that can be potential targets for plant disease manipulation.

## 7 CONCLUSIONS AND FUTURE PERSPECTIVES

We provide an in‐depth analysis of the signalling landscape of the TOR signalling network in phytopathogens and the specific inhibitors widely applied on this pathway. We discuss recent advances made in uncovering the roles of the TOR pathway in promoting phytopathogen growth and proliferation related to infecting and colonizing the host. We provide emerging roles of the plant TOR pathway in plant–phytopathogen interactions. In addition, we present disease control strategies using this pathway. As outlined in this article, recent exciting results with the pathogenic fungal TOR have improved our understanding of this pathway. Even so, we have much yet to learn. The function of upstream and downstream regulators of the TOR protein remains largely unelucidated in the fungal kingdom. Besides, the precise molecular mechanism of the TOR pathway in regulating fungal growth and virulence is uncovered, which opens new avenues for further study (Figure 4). Nowadays, CRISPR/Cas9‐based genome editing is an important tool used for crop improvement and enhancing biotic stress tolerance (Jaganathan et al., 2018). The simple designing and cloning methods and the potential ability to target multiple sites in the genome mean that CRISPR/Cas9‐based genome editing is being applied vigorously in many plant systems. However, there is no relevant application in fungal TOR studies. Targeted mutagenesis of the pathogen TOR pathway may result in the inhibition of fungal development and pathogenicity. This method could mitigate the off‐target effects caused by other therapies. Much more effort should be made to search for novel therapies to mitigate the effect of the disease caused by phytopathogenic fungi via targeting the TOR pathway. For instance, engineered nanoparticles (NPs) have been extensively applied in plant disease management (Elmer & White, 2018; Kumar et al., 2022). If possible, NPs that target the TOR pathway could be exploited to control pathogens' invasion and infection. Successful suppression of fungal disease may require an integrated approach that combines at leaast two methods, to mitigate the effects of the disease better. For example, the high costs of production associated with chemical fungicides and biopesticides, or the instability of siRNAs may limit their use in plant disease control. When combined with genetic modification technologies in host plants such as HIGSs, the dosage of these chemical fungicides and biopesticides will be reduced. We also expect that these chemical fungicides and biopesticides will be commercially available in the future. Notably, the TOR signalling pathway is evolutionarily conserved among eukaryotes. When chemical fungicides and biopesticides are applied, the off‐target effects should be noted. For example, the NPs or siRNAs used should specifically target the plant‐pathogenic fungal genome to avoid potential threats to plants or human health. Finally, more fieldwork is needed to demonstrate the efficacy of these fungicides and biopesticides. Given the current dramatically increased understanding of the pathogenic fungal TOR pathway, it is hard to be anything other than very optimistic about our future ability to improve plant disease management.

Plant fungal disease control will remain a major challenge with the global population increase and climate change. In future studies in these arenas, researchers will continue to decipher novel aspects of TOR signalling in the fungal kingdom and stimulate the development of new and broad‐spectrum TOR‐based therapies to combat infection, improve agriculture, and help feed the increasing population.

## CONFLICT OF INTEREST STATEMENT

The authors declare that they have no conflicting interests.

## Supporting information


**FIGURE S1.** Sequences and structures analysis of the fungal TOR proteins. (A) Conserved domains comparison of the TOR proteins in various fungal organisms. Each value indicates the percentage of identity with the corresponding domain sequences of ScTOR1. The number in parentheses represents the number of amino acids. (B) Comparison of the kinase domains in the fungal kingdom. (C) Phylogenetic analysis of the fungal TOR proteins. The phylogenetic tree was generated with MEGA 4.0 using the neighbour‐joining method. At, *Arabidopsis thaliana*; Bc, *Botrytis cinerea*; Bgt, *Blumeria graminis* f. sp. *tritici*; Cg, *Colletotrichum graminicola*; Fg, *Fusarium graminearum*; Fo, *Fusarium oxysporum*; Hs, *Homo sapiens*; Mo, *Magnaporthe oryzae*; Pi, *Phytophthora infestans*; Pst, *Puccinia striiformis* f. sp. *tritici*; Sc, *Saccharomyces cerevisiae*; Sp, *Schizosaccharomyces pombe*; Um, *Ustilago maydis*; Vd, *Verticillium dahliae*.

**Table S1.** List of TOR components from diverse phytopathogenic fungi.

## Data Availability

Data sharing is not applicable to this article as no new data were created or analysed.
