# Supplementary material for: The TOR signalling pathway in fungal phytopathogens: A target for plant disease control
Source: Mol Plant Pathol. 2024 Nov 7;25(11):e70024. doi: 10.1111/mpp.70024 (PMC11541241; doi:10.1111/mpp.70024)
Supplement: Supplementary file 1 — FIGURE S1. Sequences and structures analysis of the fungal TOR proteins. (A) Conserved domains comparison of the TOR proteins in various fungal organisms. Each value indicates the percentage of identity with the corresponding domain sequences of ScTOR1. The number in parentheses represents the number of amino acids. (B) Comparison of the kinase domains in the fungal kingdom. (C) Phylogenetic analysis of the fungal TOR proteins. The phylogenetic tree was generated with MEGA 4.0 using the neighbour‐joining method. At, Arabidopsis thaliana; Bc, Botrytis cinerea; Bgt, Blumeria graminis f. sp. tritici; Cg, Colletotrichum graminicola; Fg, Fusarium graminearum; Fo, Fusarium oxysporum; Hs, Homo sapiens; Mo, Magnaporthe oryzae; Pi, Phytophthora infestans; Pst, Puccinia striiformis f. sp. tritici; Sc, Saccharomyces cerevisiae; Sp, Schizosaccharomyces pombe; Um, Ustilago maydis; Vd, Verticillium dahliae. [file MPP-25-e70024-s002.docx]

**
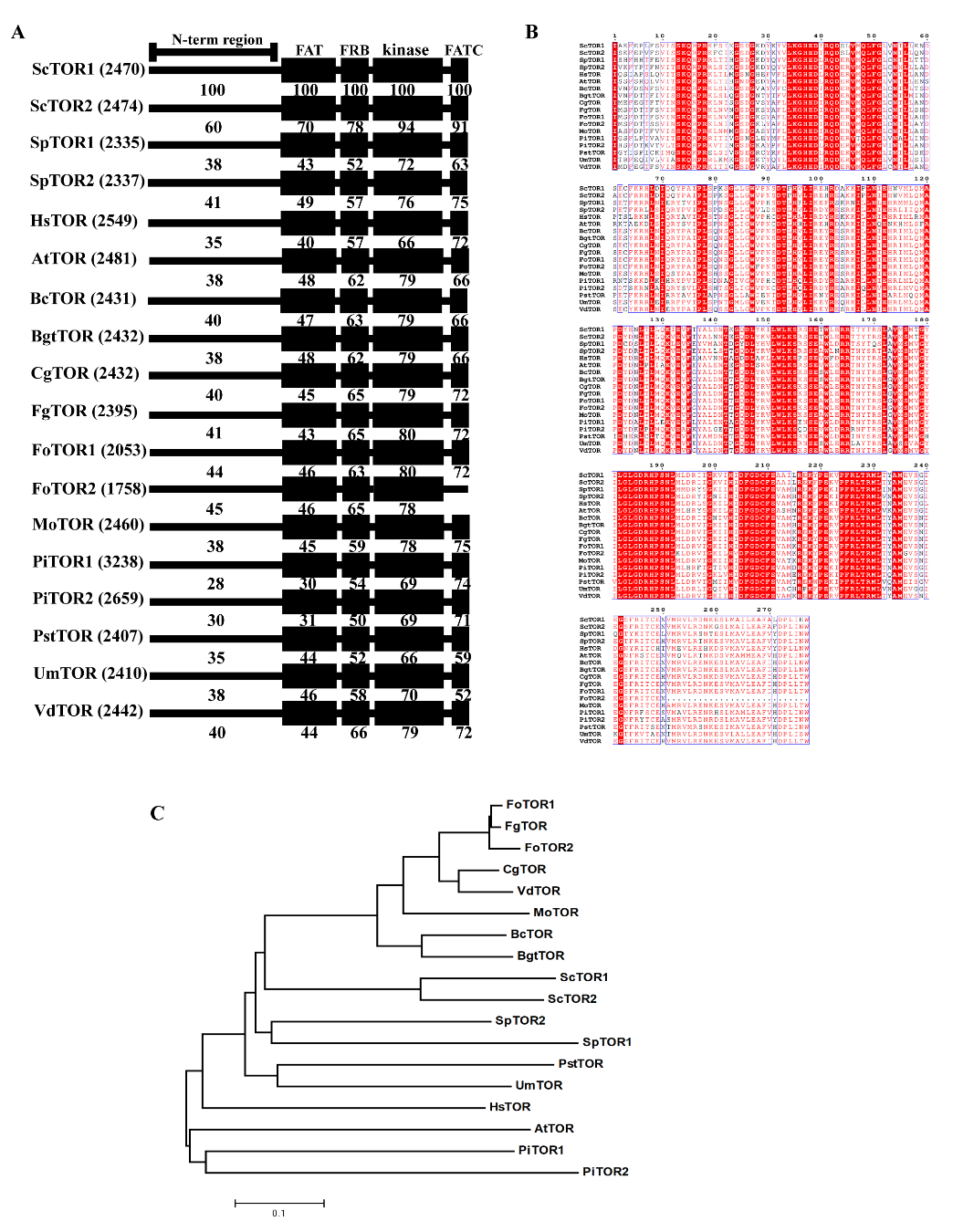
**

**FIGURE S1.** Sequences and structures analysis of the fungal TOR proteins. (A) Conserved domains comparison of the TOR proteins in various fungal organism. Each value indicates the percentage of identity with the corresponding domain sequences of ScTOR1. The number in parentheses represents the number of amino acids. (B) Comparison of the kinase domains in the fungal kingdom. (C) Phylogenetic analysis of the fungal TOR proteins. The phylogenetic tree was generated with MEGA 4.0 using the neighbor-joining method. Sc, *Saccharomyces cerevisiae*; Sp, *Saccharomyces pombe*; At, *Arabidopsis thaliana*; Hs, *Homo sapiens*; Bc, *Botrytis cinerea*; Bgt, *Blumeria graminis* f. sp. *tritici*; Cg, *Colletotrichum graminicola*; Fg, *Fusarium graminearum*; Fo, *Fusarium oxysporum*; Mo, *Magnaporthe oryzae*; Pi, *Phytophthora infestans*; Pst, *Puccinia striiformis* f. sp. *tritici*; Vd, *Verticillium dahliae*; Um, *Ustilago maydis*.
